# LncRNA MALAT1 promotes osteoarthritis by modulating miR-150-5p/AKT3 axis

**DOI:** 10.1186/s13578-019-0302-2

**Published:** 2019-07-01

**Authors:** Ying Zhang, Fuyou Wang, Guangxing Chen, Rui He, Liu Yang

**Affiliations:** Center for Joint Surgery, Southwest Hospital, The Third Military Medical University (Army Medical University), 30 Gaotanyan Main St., Shapingba Dist., Chongqing, 400038 People’s Republic of China

**Keywords:** OA, MALAT1, miR-150-5p, AKT3, MALAT1/miR-150-5p/AKT3

## Abstract

**Background:**

Many studies have reported that long noncoding RNAs (lncRNAs) could act as sponges for microRNAs (miRNAs) and play important roles in the regulation of osteoarthritis (OA). Yet, the underlying mechanisms of lncRNA metastasis-associated lung adenocarcinoma transcript 1 (MALAT1) in OA are still unclear. Therefore, we aimed to explore the regulation mechanisms of MALAT1 in OA procession.

**Methods:**

IL-1β treatment in chondrocyte was used to mimic OA in vitro. MALAT1, miR-150-5p and AKT3 expression levels were detected via qRT-PCR. The protein levels of AKT3, MMP-13, ADAMTS-5, Bax, Bcl-2, cleaved-PARP, collagen II and aggracan were measured by western blot. MTT assay was performed to detect cell proliferation ability. The apoptosis of chondrocytes was determined using flow cytometry and western blot. Luciferase assay and RNA immunoprecipitation (RIP) assays were used to confirm the relationship among MALAT1, miR-150-5p and AKT3.

**Results:**

In our study, MALAT1 and AKT3 were upregulated while miR-150-5p was downregulated in OA in vitro and vivo. The level of miR-150-5p was negatively correlated with that of MALAT1 or AKT3. More importantly, overexpression of MALAT1 promoted the expression of AKT3 by negatively regulating miR-150-5p. MALAT1 knockdown inhibited cell proliferation, promoted apoptosis, increased MMP-13, ADAMTS-5 expression and decreased collagen II, aggracan expression in IL-1β treated chondrocytes. MALAT1 upregulation or AKT3 overexpression enhanced proliferation, inhibited apoptosis and extracellular matrix (ECM) degradation, which was undermined by overexpression of miR-150-5p. By contrast, miR-150-5p depletion rescued the effect of MALAT1 downregulation or loss of AKT3 on IL-1β-stimulated chondrocytes.

**Conclusion:**

MALAT1 was responsible for cell proliferation, apoptosis, and ECM degradation via miR-150-5p/AKT3 axis.

## Background

Osteoarthritis (OA) is the most common type of joint disease and is considered be a disease involving all tissues of the joint [1–3]. The main feature of OA is the destruction of articular cartilage caused by the imbalance of extracellular matrix (ECM) components [4]. There are two main reasons for the imbalance of ECM. One is the degradation of ECM, and the other is the formation of cartilage tissue [5]. Most people over 65 and about 80% of people over 75 have OA [2]. OA can cause severe physical disability, pain, stiffness and loss of mobility, leading to obstacles in the daily activities of patients [4]. Although there are many treatments including drug treatment, acupuncture therapy, electromagnetic therapy, stem cell treatment [3, 6–13]. OA is still difficult to cure. Therefore, it is necessary to explore the deep molecular mechanisms of OA.

Many studies have shown that long noncoding RNAs (lncRNAs) are involved in cell biology processes such as cell proliferation and apoptosis, cell differentiation, tumorigenesis and metastasis [14–16]. Alterations in lncRNAs can lead to a variety of abnormal expressions of genes associated with disease and biological functions [17]. In a previous study, lncRNA FOXD2-AS1 modulated Cyclin D1 expression by sponging miR-206, resulting in regulating chondrocytes proliferation in OA [18]. LncRNA PVT1 could sponge for miR-488-3p and regulate chondrocyte apoptosis in OA [19]. LncRNA small nucleolar RNA host gene 5 (SNHG5) promotes the proliferation of chondrocytes via miR-26a/SOX2 signal axis in OA [20]. LncRNA-CIR enhances articular cartilage degeneration in OA through regulating autophagy [21]. However, the underlying mechanisms of action of lncRNA MALAT1 in OA are still lacking. MicroRNAs (miRNAs), a series of small noncoding RNAs with 18–22 nts in length, have been reported to be interacted with lncRNAs to exert its biological function [22, 23]. For example, miR-630 promotes epithelial ovarian cancer proliferation and invasion via targeting Krüppel-like factor 6 [24]. Many studies also have reported the roles of miR-150-5p. miR-150-5p is also involved in the linc00673 depletion-mediated the proliferation, migration, invasion and EMT suppressing effect in non-small cell lung cancer [25]. A previous study also indicated that miR-150-5p enhanced cell apoptosis in paclitaxel-resistant ovarian cancer cells by targeting Notch3 [26]. Although many researchers have investigated the function of miR-150-5p in many cancers [27, 28], its role in OA remains largely unknown.

In the present study, we sought to explore the function of MALAT1 and its underlying mechanisms in OA. In addition, whether miR-150-5p and AKT3 is associated with the function of MALAT1 is also addressed.

## Materials and methods

### Clinical samples of cartilage tissues

OA cartilage tissues were obtained from OA patients underwent total knee replacement surgery (n = 42, 22 men and 20 women, age range 59–70 years). Normal cartilage tissues were acquired from patients underwent the amputation without OA or rheumatoid arthritis history (n = 20, 10 men and 10 women, age range 38–47 years). All cartilage tissues conformed to the diagnostic criteria of osteoarthritis of the Orthopaedic Society of the Chinese Medical Association. Cartilage tissue samples were immediately frozen in the liquid nitrogen for storage after surgery. This study was approved by the Research Ethics Committee of Southwest Hospital, the Third Military Medical University (Army Medical University). And all the donors and their families read and signed the informed consents.

### Cell culture

The excess fibrous connective tissue in the normal cartilage tissues was subtracted, and the cartilage tissue was cut into about 1 mm^3^ size. After washing with PBS containing penicillin sodium and gentamicin, fivefold volume of 0.25% trypsin was added, digested in incubator at 37 °C for 30 min, discarded supernatant; fivefold volume of 0.2% Collagenase type II was added, digested in 37 °C incubator for 16 h, and cells were collected every 4 h. The cell suspension was filtered and digested by 200 mesh filter at 1000 r/min. After centrifugation for 5 min, the supernatant was discarded. The precipitation was washed 3 times with complete culture medium containing 10% fetal bovine serum. Finally, the cells were inoculated in a culture flask at a density of 1 × 10^5^/mL and cultured in the incubator containing 5% CO_2_ at 37 °C. Small interfering RNA for MALAT1 (si-MALAT1) and si-MALAT1 scramble (si-NC), MALAT1 overexpression plasmid or pcDNA 3.0 (NC), miR-150-5p mimic (miR-150-5p) or negative control mimic (miR-NC), miR-150-5p inhibitor (anti-miR-150-5p) or negative control inhibitor (anti-miR-NC), and si-AKT3 or AKT3 overexpression plasmid were transfected into chondrocytes. After 48 h of transfection, chondrocytes were stimulated with IL-1β (10 ng/mL; Sigma, St. Louis, MO, USA) for the 24 h and used for further analysis.

All the experiments were done with the second and third subculture of chondrocytes. All operations are performed under sterile conditions.

### qRT-PCR

Total RNAs were isolated from human cartilage tissues and cultured chondrocytes using the TRIzol reagent. Isolated RNAs were reverse-transcribed with the use of a kit (Promega). Quantitative real-time PCR (qRT-PCR) was performed on the Biosystems 7300 Real-Time PCR system (ABI, Foster City, CA, USA) by using SYBR GreenMix (Takara). ΔΔCt method was used to calculate the relative gene expression. U6 expression was used as the internal control for lncRNA and miRNA expression. β-Actin was used to normalize the mRNA expression level.

### Western blot

Total proteins were extracted by lysing cells on ice with RIPA lysis buffer (Pierce, Rockford, IL, US) for 15 min. The protein concentration was measured by the BCA Assay Kit (Thermo Scientific). Protein fractions were separated with sodium dodecyl sulfate–polyacrylamide gel electrophoresis gels (10%) and then transferred onto polyvinylidene difluoride (PVDF) membrane (Millipore, Bedford, MA, USA). After blocked with 5% BSA for 2 h at 4 °C, membranes were incubated with specific primary antibodies anti-AKT3, anti-β-actin, anti-MMP-13, anti-ADAMTS-5, anti-collagen II and anti-aggrecan (1:1000 dilution; Abcam) overnight at 4 °C. The membranes were washed thrice and then incubated with horseradish peroxidase (HRP)-conjugated secondary anti-rabbit antibodies for 1 h. All protein bands were detected by ECL detection kit (Thermo, Waltham, MA, USA) and ChemiDoc XRS System (Bio-Rad, Hercules, CA, USA). β-Actin was used as an internal normalization control.

### MTT assay

The growing conditions of cells were examined by MTT assay. The cells grown in logarithmic phase were inoculated into a 96-well culture plate at a density of 1 × 104/well, and cultured in a DMEM/F12 medium containing 10% fetal bovine serum at 37 °C in a 5% CO_2_ incubator. Each group included 5 duplicate holes. 50 μL MTT solution was added at 24, 48, and 72 h after transfection, and the culture was continued for 4 h. After discarding the supernatant, 200 μL DMSO was added. The A value was measured at 490 nm.

### Flow cytometry

1 × 10^6^ cells were washed twice with PBS. Then 200 μL binding buffer and 10 μL FITC-labeled Annexin-V (20 μg/mL) and 5 μL PI (50 μg/mL) were added, and the cells were incubated in dark at room temperature for 30 min. Then 400 μL PBS was added, and cells were analyzed by using FACScalibur cytometer and Cell-Quest Version 3.2.1 software. A tube without Annexin V-FITC and PI was used as a negative control. All experiments are repeated three times.

### Dual luciferase assay

To construct MALAT1-WT, MALAT1-MUT, AKT3-WT, AKT3-MUT luciferase reporters, 3′UTR of MALAT1-WT, MALAT1-MUT, AKT3-WT, AKT3-MUT was amplified and inserted into the pmirGLO vector (Invitrogen, Carlsbad, CA, USA). MALAT1-WT, MALAT1-MUT, AKT3-WT or AKT3-MUT and miR-150-5p, miR-NC, anti-NC or anti-miR-150-5p were co-transfected into the chondrocytes using Lipofectamine 2000 (ThermoFisher Scientific). 48 h after transfection, dual-luciferase reporter gene assays were performed to measure the luciferase activity with Dual-Luciferase Reporter Assay System kit (Promega, USA).

### Anti-Ago2 RIP assay

Chondrocytes cells were transfected with miR-NC or miR-150-5p respectively. 48 h after transfection, RIP assays were performed with the transfected cells by using the Magna RIPTM RNA Binding Protein Immunoprecipitation Kit (Millipore, Bedford, MA, USA). Then the cells were incubated with anti-Ago2 antibody (Millipore) or negative control IgG (Millipore), and the relative enrichment of MALAT1 and AKT3 were measured by qRT-PCR.

### Statistical analysis

Data were shown as the mean ± SD. Prism software was used to analysis data. Data between two groups were analyzed by using the t-test. Data from more than two groups were analyzed using the One-Way ANOVA method. All experiments were performed and analyzed in triplicate. P < 0.05 was considered significant.

## Results

### Different expression levels of MALAT1, miR-150-5p and AKT3 in normal and OA cartilage tissues

**Fig. 1:**
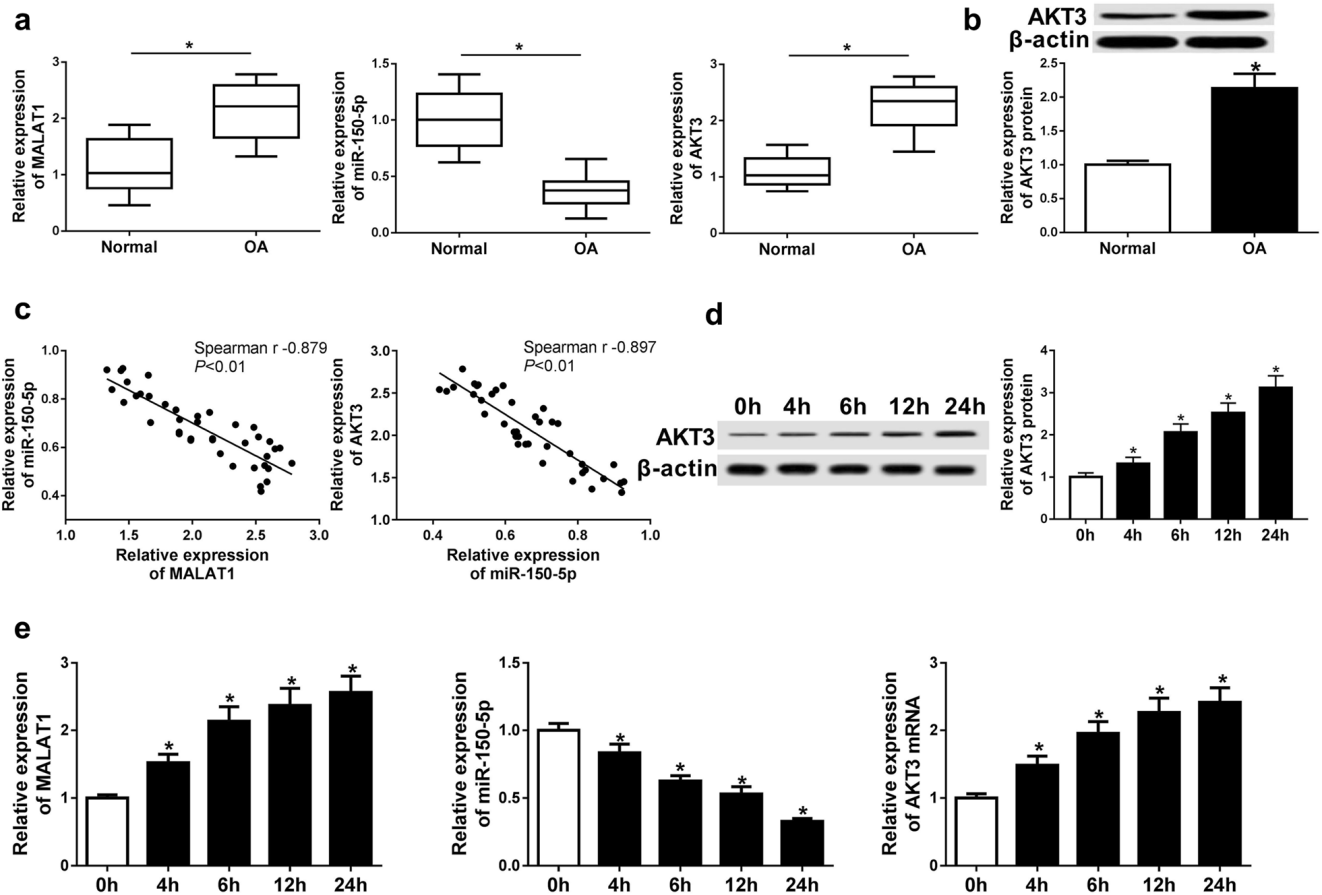
Different expression levels of MALAT1, miR-150-5p and AKT3 in normal and OA cartilage chondrocytes. **a**, **b** The expression levels of MALAT1, miR-150-5p and AKT3 in OA and normal cartilage tissues were detected by qRT-PCR and western blot. Samples were from 42 OA patients and 20 healthy subscribers. **c** Spearman was used to analyze the relationship between MALAT1, miR-150-5p and AKT3. **d**, **e** qRT-PCR and western blot were used to examine the levels of MALAT1, miR-150-5p and AKT3 in chondrocytes with treatment of 10 ng/mL IL-1β for 4, 6, 12, and 24 h. *P < 0.05

We performed qRT-PCR to examine the expressions of MALAT1, miR-150-5p and AKT3 in OA and normal cartilage tissues respectively. As shown in Fig. 1a the expressions of MALAT1 and AKT3 were significantly upregulated in OA cartilage tissues compared with that of normal tissues. On the contrary, the expression of miR-150-5p was markedly reduced in OA cartilage tissues. The western blot analysis of AKT3 was also consistent with the qRT-PCR result which displayed an increased level of AKT3 in OA cartilage tissues (Fig. 1b). In addition, to evaluate the relationship between MALAT1, miR-150-5p and AKT3, we used Spearman to analyze the correlations between them. As displayed in Fig. 1c, the expression level of miR-150-5p was negatively correlated with that of MALAT1 or AKT3. Furthermore, the chondrocytes treated by 10 ng/mL IL-1β for 4, 6, 12, and 24 h showed the increased expression of MALAT1 and AKT3 while decreased expression of miR-150-5p with the increase of treatment in time, consistent with the result in OA cartilage tissues (Fig. 1d, e). In the following studies, OA progress was induced by the treatment of chondrocytes with 10 ng/mL IL-1β. These results indicated that the expressions of MALAT1 and AKT3 were both up-regulated in OA, while the expression of miR-150-5p was down-regulated.

### MALAT1 indirectly regulated AKT3 through targeting miR-150-5p

**Fig. 2:**
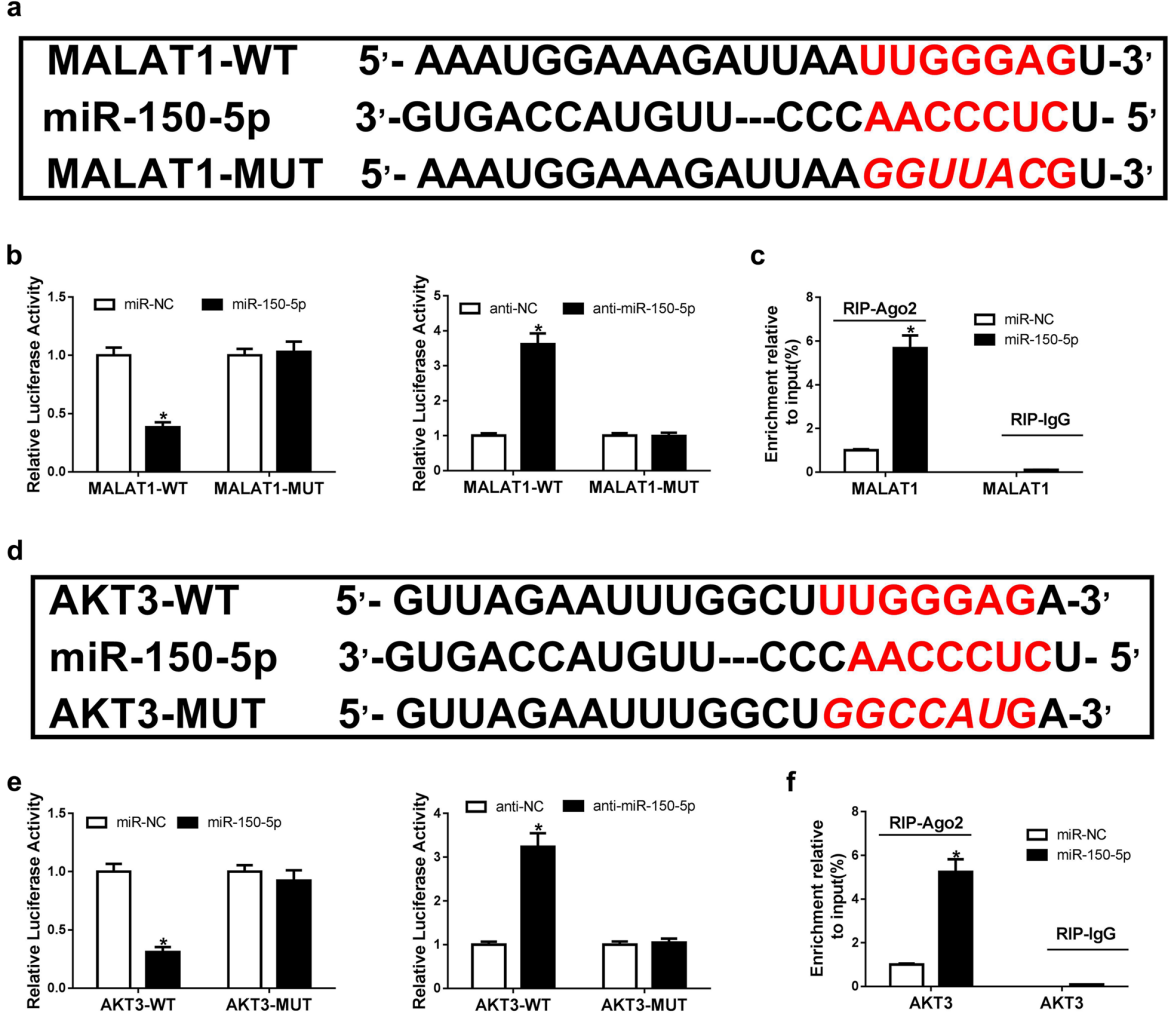
MALAT1 indirectly regulates AKT3 by targeting miR-150-5p. **a** The surmised binding sites for interaction between miR-150-5p and MALAT1. **b** Dual luciferase reporter assay was performed to conform the interaction between MALAT1 and miR-150-5p. **c** Anti-Ago2 RIP assay was carried out to identify the correlation between MALAT1 and miR-150-5p. **d** The surmised binding sites for correlation between miR-150-5p and AKT3. **e** Dual luciferase reporter assay was performed to confirm the interaction between miR-150-5p and AKT3. **f** Anti-Ago2 RIP assay was carried out to identify the correlation between miR-150-5p and AKT3. *P < 0.05

It had been found in previous studies that MALAT1 could achieve its function through correlating with miRNAs in OA progression. For further exploration of the potential mechanism of MALAT1 in OA progression, we firstly searched for the miRNAs correlated with MALAT1 by miRcode. Then, the potential miRNA binding sites in MALAT1 were predicted and miR-150-5p was selected to be the possible target of MALAT1 (Fig. 2a). Besides, the luciferase reporter assay was carried out to confirm the relationship between MALAT1 and miR-150-5p. As shown in Fig. 2b, MALAT1 was directly targeted to miR-150-5p. In addition, we took anti-Ago2 RIP assay to illuminate the endogenous relationship between MALAT1 and miR-150-5p. As suggested in Fig. 2c, MALAT1 were specifically enriched in the Ago2 pellet compared with control IgG immunoprecipitates. Moreover, we performed bioinformatics analyses (TargetScan, miroRNA.org and Starbase v2.0) to search for the potential target mRNAs of miR-150-5p and found AKT3 as a potential target (Fig. 2d). Dual luciferase reporter assays (Fig. 2e) and anti-Ago2 RIP assay (Fig. 2f) were also performed to identify the interaction of miR-150-5p and AKT3, and the results proved that AKT3 was a direct target of miR-150-5p. Thus, we thought that MALAT1 may achieve its function by directly targeting miR-150-5p, which targets AKT3 to produce influence on chondrocytes in OA.

### MALAT1 indirectly promotes AKT3 expression by competitively binding to miR-150-5p

**Fig. 3:**
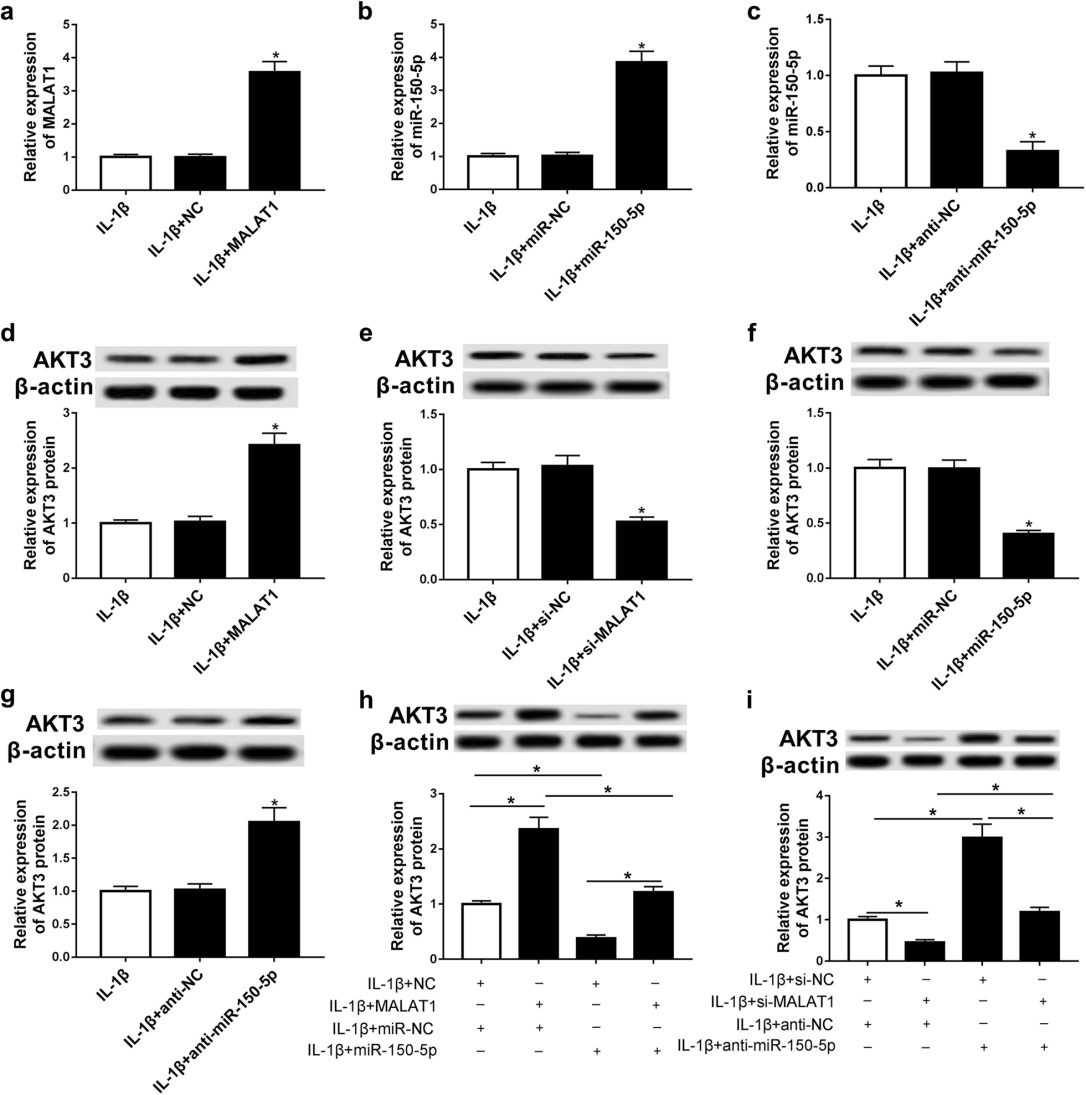
MALAT1 competitively bound to microRNA-150-5p and indirectly promotes AKT3 expression. **a**–**c** Chondrocytes were introduced with miR-150-5p mimic, miR-NC, anti-miR-150-5p, anti-miR-NC, NC, or MALAT1 before IL-1β treatment. qRT-PCR was used to measure the level of miR-150-5p. **d**–**i** Western blot analysis of AKT3 in each group. *P < 0.05

In order to further explore the regulation of MALAT1 on AKT3, MALAT1 and miR-150-5p were up-regulated or down-regulated in chondrocytes before IL-1β treatment. The expression of AKT3 was measured by qRT-PCR and western blot. The transfection efficiency revealed that MALAT1 was upregulated in IL-1β-stimulated chondrocytes after transfected with MALAT1 overexpression plasmid (Fig. 3a). Similarly, the level of miR-150-5p was elevated using miR-150-5p mimic while it was decreased using anti-miR-150-5p transfection (Fig. 3b, c) in IL-1β-stimulated chondrocytes. AKT3 protein expression level was much higher when MALAT1 was up-regulated (Fig. 3d), On the contrary, the down-regulation of MALAT1 led to a decreased expression level of AKT3 (Fig. 3e). The overexpression of miR-150-5p decreased AKT3 expression level, while the expression of AKT3 was notably increased after transfecting with anti-miR-150-5p (Fig. 3f, g). After MALAT1 and miR-150-5p were co-transfected into chondrocytes, the MALAT1 overexpression-mediated promotion of AKT3 was hampered by miR-150-5p mimic transfection (Fig. 3h). After co-transfecting chondrocytes with si-MALAT1 and anti-miR-150-5p, the suppression of AKT3 by si-MALAT1 was rescued by anti-miR-150-5p (Fig. 3i). These observations demonstrated that MALAT1 may act as a competing endogenous RNA (ceRNA) and could indirectly promote the expression of AKT3 through competitively binding to miR-150-5p.

### MALAT1 knockdown suppresses cell proliferation while induces apoptosis during OA progression

**Fig. 4:**
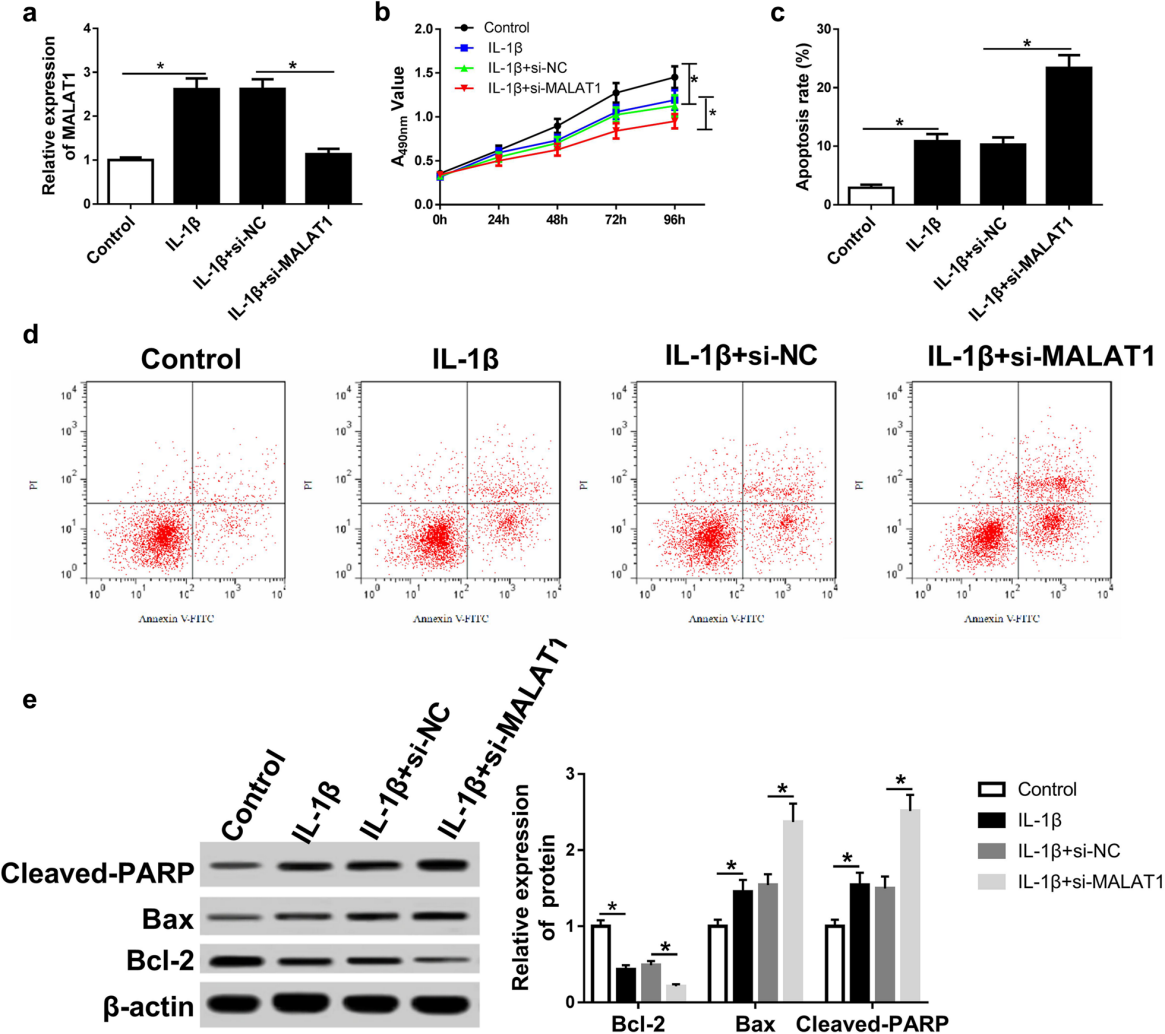
MALAT1 depletion inhibits cell proliferation and enhances cell apoptosis during OA progression. **a** qRT-PCR of MALAT1 after the transfection of si-MALAT1. 10 ng/mL IL-1β used to induce OA. **b** MTT assay was applied to measure the proliferation ability of chondrocytes after transfecting with si-MALAT1 and the stimulation of 10 ng/mL IL-1β for 24 h. **c**, **d** Apoptosis of chondrocytes was examined by flow cytometry after the same treatment in **b**. si-NC: treated with si-MALAT1 scramble; control: not treated with IL-1β. **e** Western blot was used to examine the levels of apoptosis-related protein cleaved-PARP, Bax, and Bcl-2. *P < 0.05

We treated chondrocytes with 10 ng/mL IL-1β for 24 h after transfection to investigate the influence of MALAT1 knockdown on cell apoptosis and proliferation in OA. The interfering RNA for MALAT1 was introduced into chondrocytes before IL-1β treatment. The expression of MALAT1 was significantly downregulated in IL-1β + si-MALAT1 group compared with that of IL-1β + si-NC group (Fig. 4a). MTT results demonstrated that cell proliferation was reduced in MALAT1 knockdown chondrocytes (Fig. 4b). Besides, the flow cytometry results indicated that the apoptosis of chondrocytes was increased after MALAT1 knockdown (Fig. 4c, d). Moreover, the apoptosis-related proteins cleaved-PARP, Bax, and Bcl-2 were also detected. We disclosed that anti-apoptosis-related protein Bcl-2 was downregulated, while pro-apoptosis-related protein cleaved-PARP and Bax were upregulated in IL-1β + si-MALAT1 group compared with that of IL-1β + si-NC group (Fig. 4e). These findings suggested that MALAT1 might affect the process of OA by regulating the proliferation and apoptosis of chondrocytes.

### MALAT1 depletion promotes the ECM degradation of OA chondrocytes

**Fig. 5:**
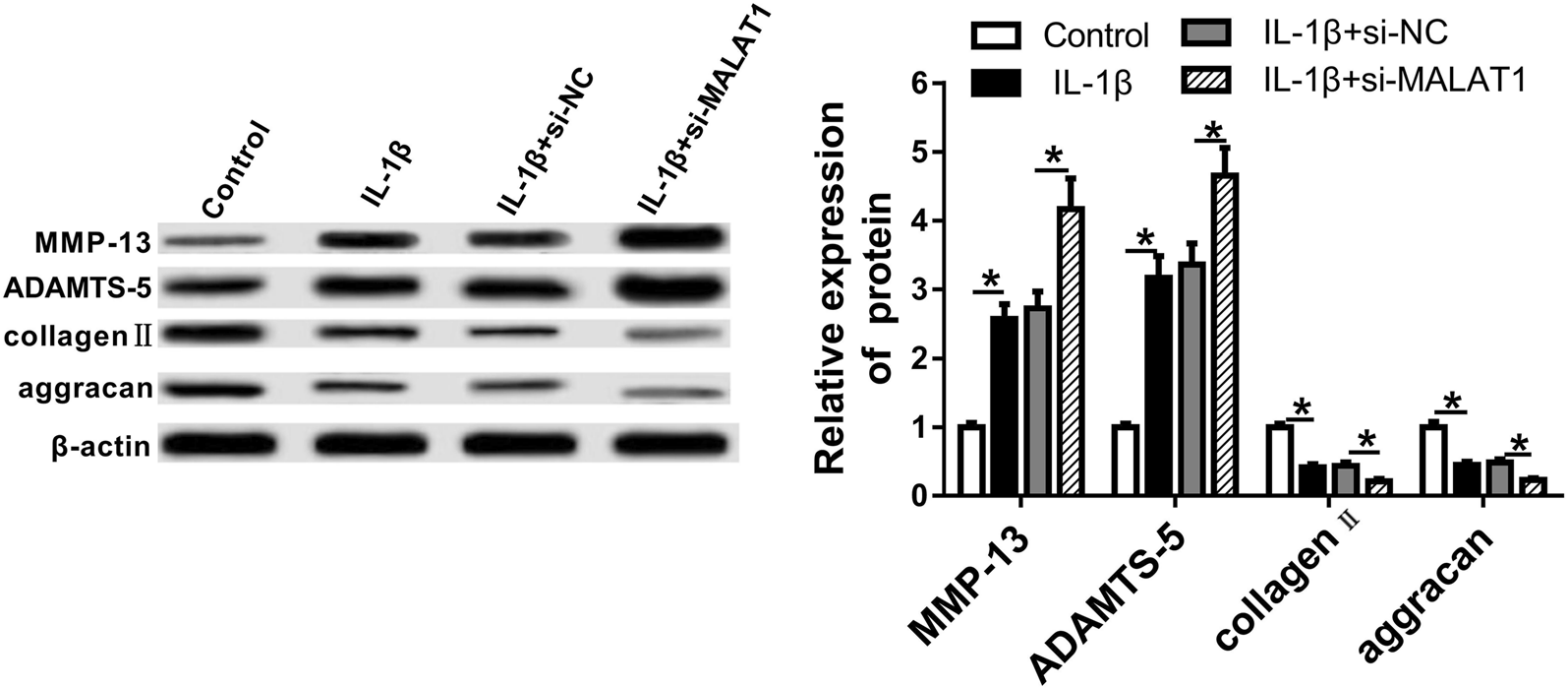
MALAT1 knockdown promotes the ECM degradation of chondrocytes in OA. The protein expression levels of MMP-13, ADAMTS-5, collagen II and aggrecan detected by western blot after transfecting with si-MALAT1 and stimulating with IL-1β (10 ng/mL) for 24 h. β-actin was used as an internal control. si-NC: treated with si-MALAT1 scramble; Control: not treated with IL-1β. *P < 0.05

To further investigate the effect of MALAT1 on IL-1β induced ECM degradation in chondrocytes, we mainly detected four critical cartilage-related genes: MMP-13, ADAMTS-5, collagen II and aggrecan. MMP-13 and ADAMTS-5 were known as major cartilage-degrading enzymes, and collagen II and aggrecan were identified as vital extracellular matrix (ECM) proteins in cartilage tissues. The western blot results in Fig. 5 displayed that the expression of MMP-13 and ADAMTS-5 were largely increased after transfecting with si-MALAT1 in OA chondrocytes. Contrarily, the protein expression levels of collagen II and aggrecan were significantly lower relative to si-NC control (Fig. 5). Taken together, these results demonstrated that MALAT1 depletion may up-regulate cartilage-degrading enzymes while down-regulate extracellular matrix proteins, thus promoting the ECM degradation of OA chondrocytes.

### MiR-150-5p reverses the influences of MALAT1 on proliferation and apoptosis in IL-1β-induced chondrocytes

**Fig. 6:**
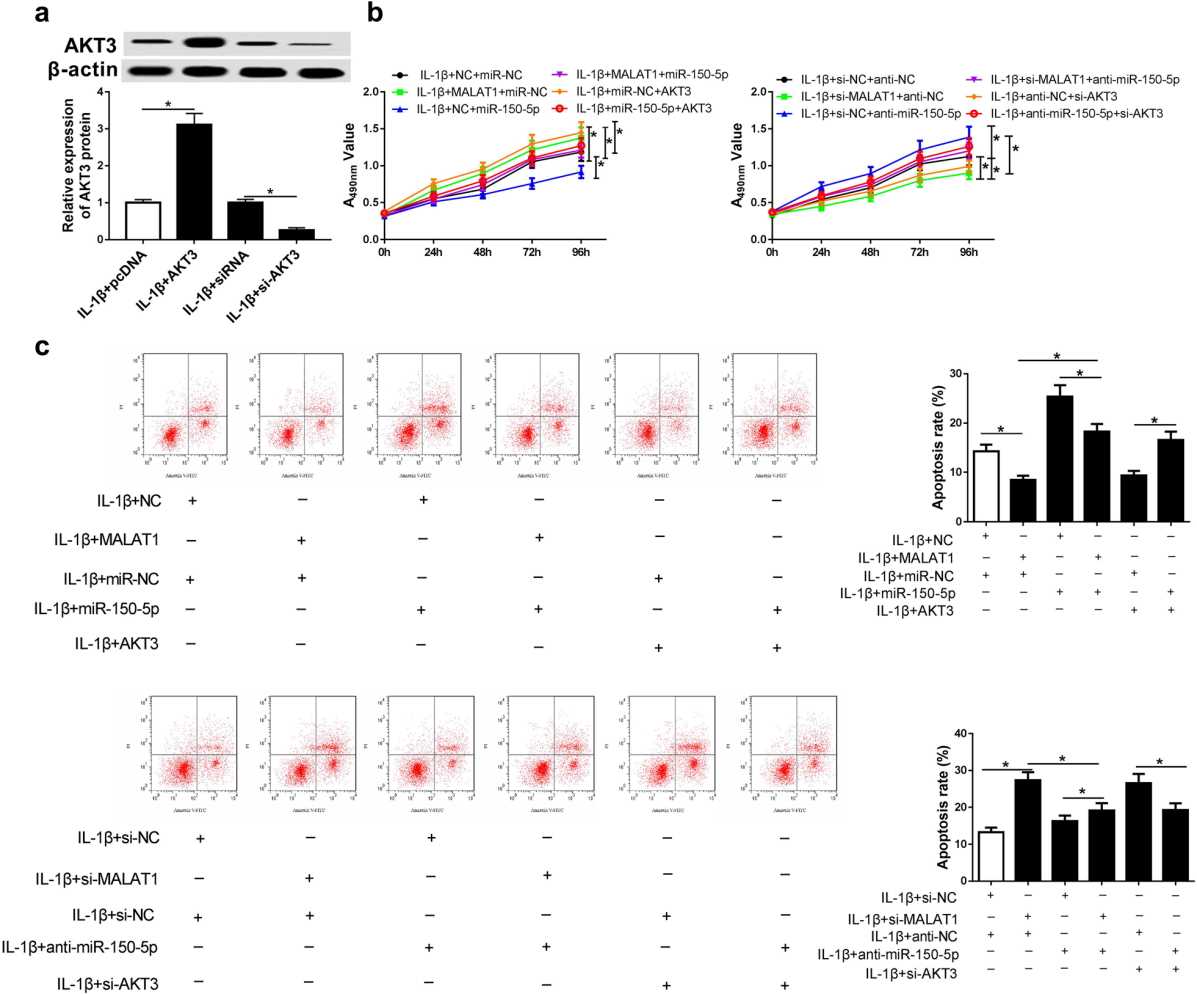
MiR-150-5p reverses the effects of MALAT1 or AKT3 on proliferation and apoptosis in OA chondrocytes. **a** Western blot was performed to detect the protein level of AKT3 in chondrocytes transfected with si-AKT3, AKT3 overexpression plasmid, or their negative controls before IL-1β treatment. **b**, **c** MTT assay and flow cytometry were performed to detect cell proliferation ability and apoptosis, respectively. Chondrocytes were transfection with NC + miR-NC, MALAT1 + miR-NC, NC + miR-150-5p, MALAT1 + miR-150-5p, miR-NC + AKT3, miR-150-5p + AKT3, anti-NC + anti-NC, si-MALAT1 + anti-NC, si-NC + anti-miR-150-5p, si-MALAT1 + anti-miR-150-5p, anti-NC + si-AKT3, and miR-150-5p + si-AKT3 before IL-1β treatment. *P < 0.05

The rescue experiments were performed to further investigate whether MALAT1 regulates cell proliferation cell apoptosis via miR-150-5p in OA. As shown in Fig. 6a, the transfection efficiency demonstrated that AKT3 overexpression plasmid or si-AKT3 could significantly induce the increase or decrease of AKT3, respectively. MTT assay was conducted to determine the proliferation ability of chondrocytes, the results shown in Fig. 6b demonstrated that up-regulation of MALAT1 or AKT3 strikingly promoted the proliferation of chondrocytes. However, this effect was dramatically blocked when miR-150-5p was overexpressed. Oppositely, cell proliferation ability was evidently suppressed in MALAT1 or AKT3 down-regulated OA chondrocytes, while the inhibition of miR-150-5p markedly reversed the effect. The results of flow cytometry were shown in Fig. 6c, overexpression of MALAT1 or AKT3 significantly inhibited the chondrocytes apoptosis while the influence was reversed by miR-150-5p overexpression. On the contrary, cell apoptosis was increased in MALAT1 or AKT3 knockdown OA chondrocytes, while the inhibition of miR-150-5p reversed the effect evidently. These results indicated that MALAT1 probably promoted proliferation and inhibited apoptosis in OA chondrocytes by competitively binding to miR-150-5p and indirectly regulating AKT3.

### MiR-150-5p reverses the effects of MALAT1 on ECM degradation of IL-1β-induced chondrocytes

**Fig. 7:**
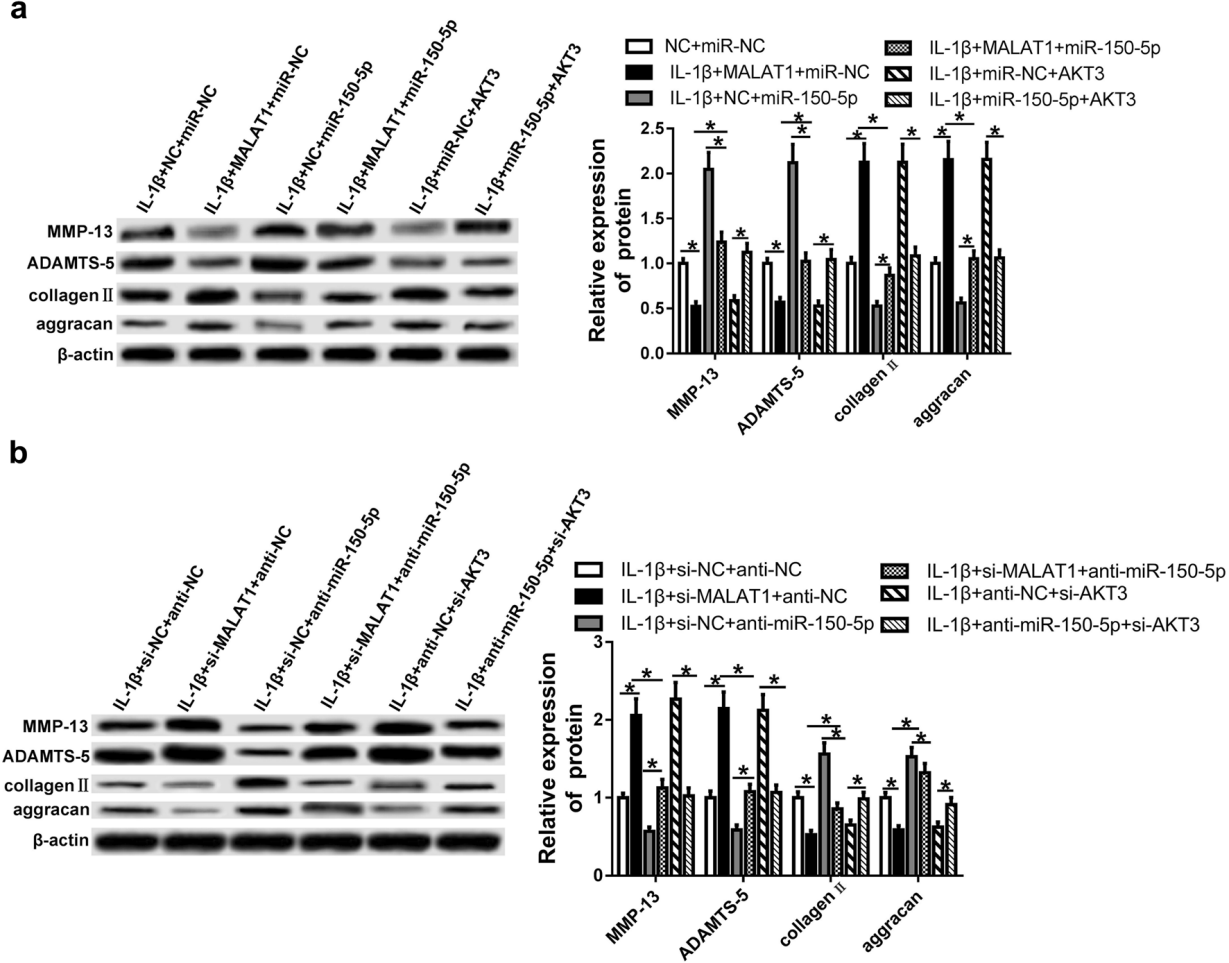
MiR-150-5p reverses the effects of MALAT1 or AKT3 on ECM degradation in OA. **a**, **b** Western blot of MMP-13, ADAMTS-5, collagen II and aggrecan after transfection with NC + miR-NC, MALAT1 + miR-NC, NC + miR-150-5p, MALAT1 + miR-150-5p, miR-NC + AKT3, miR-150-5p + AKT3, anti-NC + anti-NC, si-MALAT1 + anti-NC, si-NC + anti-miR-150-5p, si-MALAT1 + anti-miR-150-5p, anti-NC + si-AKT3, and miR-150-5p + si-AKT3 before IL-1β treatment. *P < 0.05

The rescue experiments were performed to further explore whether MALAT1 suppresses ECM degradation and facilitates cartilage formation through miR-150-5p/AKT3 axis in OA. MMP-13, ADAMTS-5, collagen II and aggrecan were detected by using western blot. When OA chondrocytes were transfected with MALAT1 or AKT3 overexpression plasmids, the protein expression levels of MMP-13 and ADAMTS-5 were decreased while the levels of collagen II and aggrecan were evidently increased. However, these effects were blocked by the overexpression of miR-150-5p (Fig. 7a). Contrarily, in MALAT1 or AKT3 knockdown OA chondrocytes, the expression levels of MMP-13 and ADAMTS-5 were significantly increased and the levels of collagen II and aggrecan were markedly reduced, while the inhibition of miR-150-5p dramatically reversed the effects (Fig. 7b). These findings demonstrated MALAT1 might inhibits ECM degradation and promotes cartilage formation via miR-150-5p/AKT3 axis in OA.

## Discussion

LncRNA MALAT1, an important lncRNA, is expressed in a variety of tissues and involved in a variety of diseases and biological processes [29]. In various cancers, the expression of MALAT1 is usually upregulated. The up-regulation of MALAT1 can enhance proliferation and inhibit apoptosis, and the down-regulation can reduce proliferation and promote apoptosis. MALAT1 expression level is significantly up-regulated in glioma stem cells, this up-regulation enhances the viability and proliferation of glioma stem cells and promotes the occurrence of glioma tumors [30]. The plasma level of MALAT1 is increased in patients with early fever after breast cancer surgery, but the inflammatory responses and metastasis of lung are decreased significantly after down-regulation of MALAT1 [31]. MALAT1 is up-regulated in human colon cancer cell lines and inhibition of MALAT1 could reduce the proliferation of colon cancer cells [32]. In another report, MALAT1 downregulation markedly represses human OA chondrocyte proliferation [33]. Consistent with previous studies, our study showed that the expression of MALAT1 was up-regulated in OA chondrocytes compared with normal chondrocytes and has a positive correlation with the severity of OA. MALAT1 knockdown in IL-1β induced chondrocytes inhibited cell proliferation while promoted cell apoptosis. Thus, we predicted that MALAT1 may have an important function in contributing to proliferation ability and restraining apoptosis in OA progression, thus promoting OA process.

Extracellular matrix ECM degradation and ECM production are two important factors affecting chondrocyte destruction [5, 34]. Members of the MMP and ADAMTS gene family play important roles in ECM degradation. Aggrecan and type II collagen are considered largely contributed to the formation of cartilage tissue [4, 35]. Our experiment measured the degradation and formation of ECM by western blot detection of MMP-13, ADAMTS-5, aggrecan and type II collagen. The down-regulation of MALAT1 increased MMP-13 and ADAMTS-5 while decreased collagen II and aggrecan in OA. Those data indicated that depletion of MALAT1 may contribute to ECM degradation and play a key role in the pathogenesis of OA. Previous studies have reported that MALAT1 could achieve its function through correlating with miRNAs [36–38]. For example, Lei et al. reported that MALAT1 participates in the ovarian cancer growth by sponging miR-506 [36]. Chang et al. also disclosed that MALAT1 could act as a ceRNA to regulate signal transducer and activator of transcription-3 (STAT3) in oral squamous cell carcinoma [37]. In this study, we found that miR-150-5p may be act as the possible target of MALAT1 by miRcode. Subsequently, we further disclosed that AKT3 was a direct target of miR-150-5p. Moreover, MALAT1 increased the expression of AKT3 by sponging miR-150-5p. Interestingly, A low expression of miR-150-5p and high expression of AKT3 were also observed in OA cartilage tissue. Therefore, we thought that MALAT1 may play important roles in OA progression by miR-150-5p/AKT3.

**Fig. 8:**
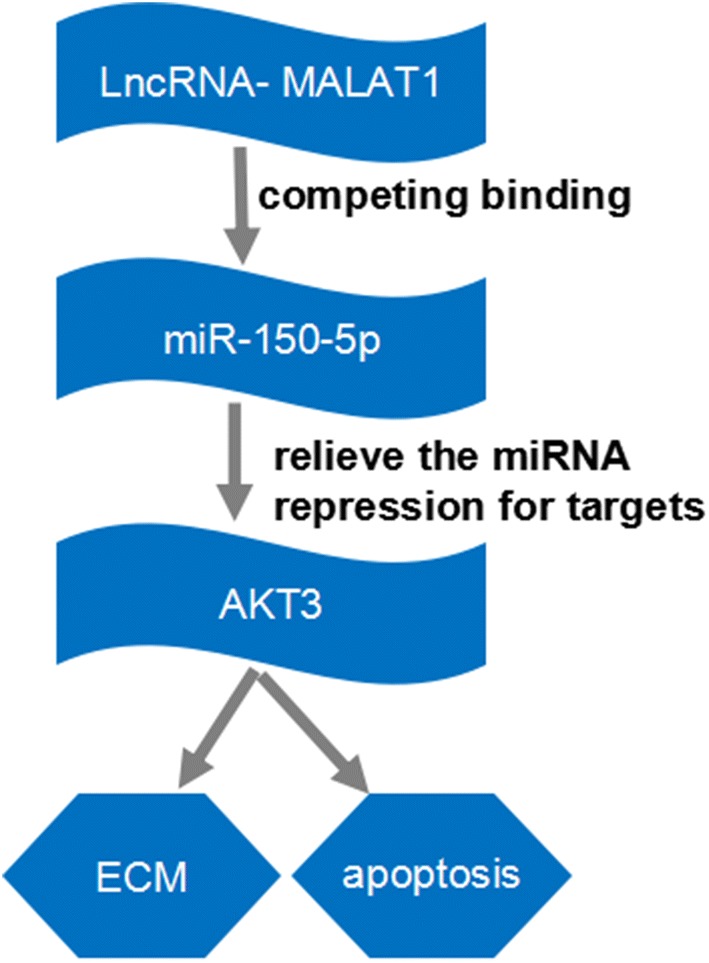
The schematic diagram of in MALAT1/MiR-150-5p/AKT3OA axis in OA. Lnc RNA MALAT1 could be act as a ceRNA of miR-150-5p to regulate the expression of AKT3, thus modulating the ECM degradation and cell apoptosis

A series studies have found that AKT was important in growth, proliferation and metabolism and MALAT1 might influenced the proliferation and metastasis of cells by regulatin*g* AKT [39–44]. Dong et al. revealed that MALAT1 could inhibit proliferation and invasion through PI3K/AKT signaling pathway [39]. AKT3 inhibition has been reported to contribute to cell apoptosis in embryonic stem cells [45]. Researchers have found that AKT3 is elevated in OA chondrocytes [46]. But, the involvement of AKT3 in MALAT1-mediated function in OA remain largely unknown. In addition, many studies have demonstrated that miR-150-5p could suppress tumor cell proliferation and migration, facility tumor cell apoptosis, and inhibit the process of tumor [47–49]. For instance, miR-150-5p curbs cell proliferation, migration, invasion and angiogenesis in colorectal cancer [48]. Recently, miR-150-5p was found to be downregulated in OA patients [50]. Yang et al. further disclosed that miR-150-5p is elevated in Interleukin-1-mediated human chondrogenic cells ATDC5 and loss of miR-150 could alleviate IL-1-induced cell damage in ATDC5 cells [51]. However, whether miR-150-5p is involved in the function of MALAT1 in OA has not been reported. In this report, we demonstrated that overexpression of MALAT1 inhibited cell apoptosis while enhanced proliferation, which was rescued by upregulation of miR-150-5p. Similarly, the depletion of miR-150-5p also attenuated the effect of absence of MALAT1. More importantly, the AKT3 also weakened the effect of miR-150-5p on cell proliferation and apoptosis. Those data suggested that MALAT1 may modulate the cell proliferation and apoptosis by miR-150-5p/AKT3 axis. Further rescue experiments found that miR-150-5p reversed the influences of MALAT1 on ECM degradation of IL-1β-induced chondrocytes. Besides, AKT3 also undermined the effect of miR-150-5p in IL-1β-induced chondrocytes, further suggesting that MALAT1 may be responsible for the cell proliferation, apoptosis and ECM degradation by miR-150-5p/AKT3 axis (Fig. 8).

## Conclusion

Taken together, we provide the evidence that MALAT1 contributed to OA progression by promoting cell proliferation and cartilage formation, and inhibiting cell apoptosis and ECM degradation. And the underlying regulatory mechanism is that MALAT1 exited its function through acting as a sponge for miR-150-5p and indirectly regulating AKT3, thus forming a MALAT1/miR-150-5p/AKT3 axis to participate in OA progression. The regulatory mechanism indicated that MALAT1 might be used as a new drug target for the treatment of OA.

## Data Availability

Not applicable.

## Abbreviations

OA: osteoarthritis
lncRNAs: long non-coding RNAs
miRNAs: microRNAs
qRT-PCR: quantitative real-time PCR
PVDF: polyvinylidene difluoride
